# Functional and Potential Therapeutic Implication of MicroRNAs in Pancreatic Cancer

**DOI:** 10.3390/ijms242417523

**Published:** 2023-12-15

**Authors:** Amartya Pal, Anushka Ojha, Jingfang Ju

**Affiliations:** 1 Department of Pathology, Renaissance School of Medicine, Stony Brook University, Stony Brook, NY 11794, USA; amartya.pal@stonybrook.edu (A.P.); anushka.ojha@stonybrook.edu (A.O.); 2 Graduate Program in Molecular and Cellular Biology, Stony Brook University, Stony Brook, NY 11794, USA; 3 The Northport Veteran’s Administration Medical Center, Northport, NY 11768, USA

**Keywords:** miRNA, pancreatic cancer, resistance, therapeutics, biomarker

## Abstract

The alarmingly low five-year survival rate for pancreatic cancer presents a global health challenge, contributing to about 7% of all cancer-related deaths. Late-stage diagnosis and high heterogeneity are the biggest hurdles in treating pancreatic cancer. Thus, there is a pressing need to discover novel biomarkers that could help in early detection as well as improve therapeutic strategies. MicroRNAs (miRNAs), a class of short non-coding RNA, have emerged as promising candidates with regard to both diagnostics and therapeutics. Dysregulated miRNAs play pivotal roles in accelerating tumor growth and metastasis, orchestrating tumor microenvironment, and conferring chemoresistance in pancreatic cancer. The differential expression profiles of miRNAs in pancreatic cancer could be utilized to explore novel therapeutic strategies. In this review, we also covered studies on recent advancements in various miRNA-based therapeutics such as restoring miRNAs with a tumor-suppressive function, suppressing miRNA with an oncogenic function, and combination with chemotherapeutic drugs. Despite several challenges in terms of specificity and targeted delivery, miRNA-based therapies hold the potential to revolutionize the treatment of pancreatic cancer by simultaneously targeting multiple signaling pathways.

## 1. Introduction

Pancreatic cancer remains a most formidable challenge, as the third leading cause of cancer-related mortality [1]. Pancreatic ductal adenocarcinoma (PDAC) is among the more aggressive subtypes of pancreatic cancer, and is typically diagnosed at a later stage, further compounding the problem [2,3]. The genetic and phenotypic heterogeneity of the disease stand as the biggest challenges in the development of efficient treatment strategies [4,5].

Despite relentless efforts to improve the management of PDAC, the five-year survival rate for pancreatic cancer remains as low as 12% [6]. Owing to these alarming statistics, as well as the limited treatment options and dismal survival rates, there is a compelling need to develop novel diagnostics and therapeutic strategies.

Among the standard treatment options, if the tumor is resectable, surgery is the most preferred path. Radiation therapy and chemotherapy are often used in combination for enhanced efficacy in metastatic tumors. Systemic chemotherapy, such as FOLFIRINOX (5-fluorouracil, folinic acid (leucovorin), irinotecan, and oxaliplatin) or gemcitabine in combination with paclitaxel, is the choice for patients with advanced disease [7,8]. Targeted therapies such as tyrosine kinase inhibitors (TKIs) are also being used to improve patient outcomes. However, one of the major limitations of chemotherapy is that advanced-stage cancers develop resistance against most of the drugs, rendering them ineffective [9,10]. To address this issue, miRNAs have emerged as promising alternatives as future therapeutic candidates for PDAC. The ability of miRNAs to target the expression of multiple genes at once, thereby allowing crosstalk between various signaling pathways, provides an attractive ground for the development of miRNA-based therapeutics that would be able to overcome resistance [11,12,13].

miRNAs are a family of short, 18–24 nucleotide, single-stranded, non-coding RNA molecules that are able to suppress the expression of genes post-transcriptionally by recognizing the 3′ untranslated region (UTR) of target messenger RNA (mRNA) [14]. These molecules can serve as invaluable biomarkers for diagnostics as well as prognostics, due to their stability in circulation and correlation with disease progression. There are several advantages of using miRNA as a biomarker vs. mRNA. The superior stability of miRNAs compared to mRNA has been established in formalin-fixed paraffin-embedded (FFPE) samples. The use of miRNAs therefore, allows for a large retrospective biomarker discovery and validation studies using a wide collection of FFPE specimens. In addition, the number of miRNAs is much smaller than that of mRNAs, which further reduces the candidate pool size. Additionally, miRNAs play an important role in regulating multiple biological processes as well as tumor targets, and their expression patterns correlate with the disease status [15,16,17]. The dysregulation of miRNA has been implicated in most cancer types and can be linked to the various hallmarks of cancer [18,19,20]. This dysregulation could be caused by chromosomal abnormalities, epigenetic alterations such as the hypermethylation of CpG islands, dysregulation in transcriptional regulation, or even direct interactions between two miRNAs, leading to the suppression of miRNA [21,22]. For example, miR-21 was found to be more abundant in pancreatic tumors compared to non-neoplastic pancreatic tissue and can be used as a prognostic marker for pancreatic cancer. Conversely, miR-192-5p and miR-194-5p play pivotal roles in downregulating genes specific to the basal subtype in the classical subtype. These miRNAs are generally underexpressed in basal subtypes, indicating their significance in subtype-specific gene regulation. Consequently, they can serve as biomarkers for discerning tumor subtypes in pancreatic cancer [23,24].

Based on their expression and distinctive roles, miRNAs can be classified into two subtypes: miRNAs with tumor-suppressor functions and miRNAs with oncogenic functions. Tumor-suppressive miRNAs are generally downregulated in cancer cells, thereby allowing for an expansion in the activity of their target genes, in this case, an oncogene. miRNAs with an oncogenic function, on the other hand, are often upregulated in cancer cells, thereby more strongly inhibiting target tumor-suppressors [25]. They have been named “oncomiRs” or “tumor suppressor miRs”; however, only a few have been fully functionally validated as true tumor-suppressor or oncogene-like miRs.

This review will cover the current literature and the latest advancements in the role of miRNAs as key players in the post-transcriptional regulation of the gene expression of pancreatic cancer and their potential role in the diagnosis and treatment of PDAC.

## 2. miRNAs in Pancreatic Cancer

**Figure 1:**
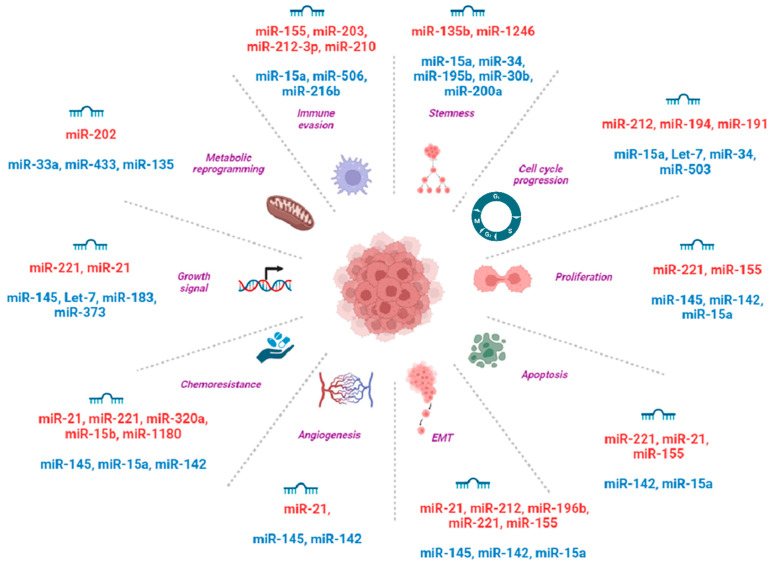
Schematic illustration of the microRNA-based regulation of the different properties of pancreatic cancer (tumor-suppressor miRNAs in blue and OncomiRs in red). Created with BioRender.com.

MicroRNAs (miRNAs) have emerged as a significant focus of research in the field of pancreatic cancer in the last decade. The aberrant expression of miRNAs has been implicated in all major cancer types, including pancreatic cancer [26]. The dysregulation in miRNA expression resulted in various hallmarks of cancer, such as uncontrolled cell proliferation, invasion, angiogenesis, metastases, and evading tumor-suppressors (Figure 1) [27,28,29,30,31]. Thus, analyzing the differential regulation of miRNA expression would provide an insight into the diagnosis and prognosis of, as well as novel therapeutic strategies against, pancreatic cancer.

With respect to cancers, the expression patterns of miRNAs are significantly altered, such that they can act as diagnostic biomarkers. For example, circulation miR-107 has been identified as a potential differentiating marker for PDAC [32]. In the context of pancreatic cancer, there are distinctive miRNA expression profiles, called the ‘miRNAome’, that can be used to distinguish between normal and cancerous tissues. miRNAs are relatively stable in blood circulation, thus making blood-based screening a potential diagnostic approach [33,34].

As we go further into the review, we will outline the role of miRNAs in pancreatic cancer. The distinctive expression profiles of different miRNAs not only provide candidate miRNAs for the early detection of pancreatic cancer but also establish novel therapeutic targets for improved patient outcomes in the face of this challenging malignancy.

### 2.1. miRNAs with Tumor-Inhibitory Functions

A number of miRNAs have been found to be downregulated in pancreatic cancer. These miRNAs, when reduced or lost in expression, exert profound effects on multiple signaling pathways, including PI3K, NF-kB, and Hedgehog, ultimately fostering the progression and metastasis of pancreatic cancer (Table 1). Some of them have been shown to fit the classical definition of tumor-suppressor miRNAs; however, many of them lack direct experimental evidence that they are truly tumor-suppressors.

One such tumor-suppressor miRNA in pancreatic cancer, miR-15a, has reduced expression in pancreatic cancer samples compared to non-cancerous tissues [35]. Although miR-15/16 has been demonstrated to be a tumor-suppressor in CLL, we only found reductions in the expression of miR-15a in pancreatic cancer [36]. miR-15a was also found to target several genes which have critical cellular functions, such as BMI-1, BCL2, Yap-1, and DCLK1, and affect different essential cellular pathways [37,38].

MiR-142, another anti-tumorigenic miRNA, is also downregulated in pancreatic cancer and inhibits proliferation as well as promoting apoptosis by regulating RAP1A [39]. This tumor-suppressive effect is mediated by targeting HIF-1α. HIF-1α is the oxygen-regulated subunit of HIF-1 and induces tumor angiogenesis. The hypoxic environment causes miR-142 to be further downregulated, while HIF-1α is upregulated, promoting hypoxic-induced cell proliferation and invasion [40]. Also, it has been shown to inhibit pancreatic cancer cell migration and invasion through the downregulation of FAK and MMP9 expression, also inhibiting phosphoinositide 3-kinase (PI3K)/AKT signaling pathway via the direct targeting of PIK3C [41]. It was reported that miR-145 regulated different targets, including MUC13, TGF-β receptor and SMAD2, and slowed pancreatic cancer progression by impeding the proliferation, migration, and invasion of cancer cells [42,43,44]. Wang et al. demonstrated that miRNA-145 has the ability to prevent cancer cell invasion and angiogenesis by downregulating angiopoietin-2 [45].

Epithelial–mesenchymal transition (EMT) is one of the most important hallmarks of cancer, regulated by a group of EMT-activating transcription factors (EMT-TFs). In the context of pancreatic cancer, ZEB1 stands out as a significant contributor to the EMT because of its ability to seamlessly transition between a transcriptional repressor and activator, depending on how it interacts with co-activators like Lef1, YAP1, P300, and Smad [46,47]. Multiple studies have unveiled the participation of various miRNAs, including let-7 and miR-183, in the regulation of the Hippo pathway, a pivotal player in cancer development and metastasis. One such example is LATS2, a direct target of miR-373, which promotes tumor growth when downregulated, signifying the substantial influence of miRNAs on the Hippo pathway [48,49,50]. Let-7 also inhibits the progression of pancreatic cancer cells by upregulating the suppressor of cytokine signaling 3 (SOCS3) expression, leading to the inhibition of STAT3 phosphorylation [51]. miR-503 was shown to bind the 3′UTR of CCND1 mRNA (encodes for Cyclin D1, a crucial regulator of the cell cycle) and inhibit the proliferation of PDAC cells [52].

Numerous other tumor-suppressive miRNAs, including miR-383, miR-375, miR-216b, miR-455, miR-202, and miR-494, were shown to be downregulated in the pancreatic cancer landscape. The underexpression or loss of expression of these miRNAs plays a substantial role in the progression of the disease by impacting multiple signaling pathways, such as AK4P1/SP1, c-MYC/SIRT1, the Wnt/β-catenin pathway, and the DLEU2/SMAD2 pathway, that lead to cancer cell proliferation, invasion, metabolism, and immune responses [53,54,55,56,57]. In the complex landscape of pancreatic cancer, these miRNAs bear promise as prognostic markers and potential targets for therapeutic intervention.

In summary, in the context of pancreatic cancer, the downregulation of these many tumor-suppressive miRNAs exerts a substantial influence over multiple signaling pathways. This intricate network ultimately drives the development and metastasis of pancreatic cancer. Delving into the complex relationships between these miRNAs and associated cellular pathways is crucial for the advancement of targeted therapies for this formidable disease.

### 2.2. miRNAs with Oncogenic Functions

In addition to tumor-suppressive miRNAs, several miRNAs are upregulated in PDAC. These miRNAs often play crucial roles in promoting the carcinogenic process, malignant transformation, and metastasis (Table 1). They hold the capacity to enhance cancer progression by suppressing the expression of genes involved in the regulation of cell proliferation and cell cycle progress via the repression of mRNA translation or destabilization [58].

One such miRNA in PDAC is miR-212, which is up-regulated in PDAC samples and cells. miR-212 promotes PDAC progression and metastatic ability through its modulation of PTCH1 expression [59]. miR-196b correlates with poor differentiation of cancerous cells, larger tumor dimensions, lymph node invasion, and advanced TNM staging. It directly targets the cell adhesion molecule, CADM1, which is involved in cell adhesion and apoptosis. Thus, miR-196b can play a crucial role in regulating PDAC progression [60,61].

Additionally, miR-221-3p, another up-regulated miRNA in PDAC, is overexpressed in PDAC and has been associated with increased cell proliferation and reduced apoptosis, and its circulating levels correlate with distant organ metastasis and advanced TNM staging [62,63]. miR-10b consistently stands out as one of the most up-regulated miRNAs in PDAC cells. Low levels of miR-10b have been associated with a better response to neoadjuvant therapy, a higher likelihood of surgical resection, delayed metastasis, and improved patient survival, underscoring its importance in PDAC [64]. Another upregulated miRNA, miR-155, could also downregulate the expression of the suppressor of cytokine signaling (SOCS1) to increase the phosphorylation of STAT3 and promote the proliferation and invasion of pancreatic cancer cells [65,66]. 

Several other miRNAs were found to be overexpressed in cancer cells, including miR-301a-3p, miR-205, and miR-21. They were found to play pivotal roles in regulating key signaling pathways in PDAC. The up-regulation of miR-301a-3p is accompanied by the downregulation of SMAD4 in pancreatic cancer cells, resulting in increased tumorigenicity, invasion, and migration [67,68]. miR-21, a widely reported oncomiR found to be overexpressed in various cancer types, inhibits cell proliferation, and promotes cell death by apoptosis, revealing its critical role in driving PDAC progression. In their study, Sun et al. suggested that a reduction in miR-21 enhances PTEN tumor-suppressor expression in pancreatic cancer as well as leading to the suppression of pancreatic cancer cell proliferation by inhibiting the HIF-1α/VEGF signaling pathway and downregulating matrix metalloproteinase 2 (MMP-2) and MMP-9 expression [69]. miR-483-3p has been found to target SMAD4, which influences cell survival, apoptosis, and differentiation [70]. Additionally, miR-23a has been implicated in downregulating APAF1, TFPI-2, and epithelial splicing regulatory protein 1, contributing to pancreatic ductal cancer development [71,72,73].

In summary, miRNAs with oncogenic functions are instrumental in promoting PDAC progression by targeting specific signaling pathways. Understanding their roles and mechanisms of action can offer insights into potential therapeutic targets for this aggressive cancer.

### 2.3. MicroRNAs in Chemoresistance

Another important aspect of the treatment of pancreatic cancer is chemoresistance. As miRNAs can impact diverse cellular pathways, they possess the potential to regulate the chemoresistance of pancreatic cancer cells. There are several pro-tumorigenic miRNAs that promote resistance to various chemotherapeutic drugs.

5-fluorouracil (5-FU), the first approved and first-line chemotherapeutic drug for pancreatic cancer, is still widely used to treat several pancreatic cancer cases [74]. However, inherent and acquired drug resistance is the major challenge to the success of 5-FU chemotherapy in treating pancreatic cancer. Multiple research projects have shown that several pro-tumorigenic (such as miR-21, miR-221, and miR-320a) or tumor-suppressor miRNAs are associated with 5-FU resistance in pancreatic cancer. An elevated level of miR-21 was shown to promote drug resistance to 5-FU and enhance the proliferation of pancreatic cancer cells by downregulating the expression of its target genes PTEN and PDCD4 [75]. Similarly, miR-320a was found to be upregulated in 5-FU-resistant pancreatic cancer cells, causing induced mesenchymal phenotype, enhanced cell invasion and migration, and 5-FU resistance by binding to the 3′UTR of PDCD4 mRNA in pancreatic cancer [76]. Also, the overexpression of miR-221-3p contributes to increased 5-FU resistance by directly binding to the 3′UTR of RB transcriptional corepressor 1 (RB1) and downregulating its expression [77]. In pancreatic cancer cells, miR-146a-5p exhibits significant upregulation and plays a role in regulating pancreatic cancer development and resistance to chemotherapy, primarily by suppressing the TRAF6/NF-κB p65/P-gp axis [78]. 

Gemcitabine, a nucleoside analogue of deoxycytidine, is normally used in combination with other drugs to treat several solid tumors. However, recent studies have shown an increase in Gemcitabine resistance and poor prognosis in pancreatic cancer. Several miRNAs can cause apoptosis evasion in pancreatic cancer cells, which is one of the primary causes of gemcitabine resistance in pancreatic cancer. For example, miR-155, miR-17-5p, and miR-210 can regulate different effectors of apoptosis, such as the downregulation of pro-apoptotic proteins like Bim or overexpression of anti-apoptotic proteins like Bcl-2 [79]. Also, the downregulation of miR-140-3p causes oncogenic WNT5A expression and increases stem cell features in PDAC cells, resulting in Gemcitabine resistance in PDAC [74]. There are some other miRNAs which are upregulated in tumor cells, like miR-223 and miR-15b, which might play a role in potentiating Gem resistance via EMT, while others, like miR-21, miR-221, and miR-301, induce Gem resistance by targeting PTEN [80,81]. miRNAs like miR-1180 promote oncogenic processes by directly regulating TNIP2, a suppressor of NF-κB signaling, and causing cisplatin resistance [82]. 

### 2.4. miRNAs in Pancreatic Cancer Stem Cells

Pancreatic stellate cells (PSCs) play a pivotal role in establishing a niche for cancer stem cells (CSCs) in pancreatic cancer by secreting embryonic morphogens, including Nodal/Activin [83]. This intricate interplay between PSCs and CSCs underscores the significance of stromal cells in shaping the tumor microenvironment and promoting aggressive cancer behavior [84].

Multiple miRNAs are involved in the regulation of pancreatic CSCs. For example, miR-15a functions as a tumor-suppressor by downregulating the stemness marker BMI-1 [37]. miR-34 targets key oncogenic factors like c-Met and influences the expression of CSC markers, such as CD44 and CD133, thereby inhibiting tumor formation and enhancing the sensitivity of pancreatic cancer cells to certain chemotherapies [85]. Anti-miR-135b has also been shown to decrease the expression of critical stem cell markers, including NANOG, ALDH1, SOX2, and OCT-4, both in vitro and in vivo, highlighting its potential as a therapeutic tool [86]. MiR-195 plays a role in downregulating DCLK1, which is involved in the maintenance of pancreatic CSCs [87]. MiR-30b plays a crucial role in reducing migration and invasion by reversing EMT, and inhibits tumorigenicity in PSCs that are CD24-, CD44-, and EpCAM-triple-positive and exhibit stemness characteristics [88].

Furthermore, miRNAs belonging to the miR-200 family, such as miR-200a, play a role in regulating stemness in pancreatic cancer by reducing the expression of CSC markers and EMT-related genes such as CD24, CD44, EpCAM, as well as the EMT markers N-cadherin, ZEB1, and vimentin [89]. The interconnected roles of these miRNAs and the gene DCLK1 underscore their importance in pancreatic cancer progression. Additionally, miR-205 is downregulated in pancreatic cancer and its overexpression decreases the expression of both general stemness markers such as OCT-3/4 and NANOG and more specifically pancreatic CSC markers, including CD44 and ALDH1 [90].

In contrast to tumor-suppressor miRNAs, miR-1246 is generally overexpressed in pancreatic cancer cells and correlated with a poor prognosis in pancreatic cancer. This upregulation is associated with an increased population of CD44+/CD24+ cells, enhanced sphere-forming ability, and the enrichment of stemness pathways, contributing to drug resistance to gemcitabine [91]. 

In conclusion, the dysregulation of several stemness genes like TGF-β and DCLK1 in pancreatic cancer cells and pancreatic stellate cells by various miRNAs collectively contribute to the maintenance and activation of pancreatic cancer stem cells, influencing the disease’s aggressiveness, progression, and therapeutic response.

### 2.5. miRNAs in Tumor Microenvironment and Immune Infiltration

In the context of pancreatic cancer, miRNAs play a multifaceted role in shaping the tumor microenvironment and immune infiltration. One crucial aspect is the involvement of extracellular vesicles (EVs) that transport miRNAs and other biomolecules, impacting various disease processes. Several miRNAs, such as miR-21, miR-17-5p, miR-23b-3p, miR-191, and miR-451, are carried by EVs and have emerged as potential biomarkers for pancreatic ductal adenocarcinoma (PDAC) diagnosis and prognosis [92]. These miRNAs regulate processes in recipient cells, affecting cancer progression.

Pancreatic stellate cells (PSCs) significantly contribute to the tumor microenvironment. EVs produced by PSCs are enriched with specific miRNAs, including miR-21-5p, miR-451a, miR-221, and miR-5703. These miRNAs play roles in promoting cancer cell migration, epithelial-to-mesenchymal transition (EMT), drug resistance, and cell proliferation, collectively contributing to the aggressive nature of PDAC [93].

The crosstalk between cancer-associated fibroblasts (CAFs) and cancer cells, mediated through EVs, influences the tumor microenvironment. This interaction impacts various aspects of tumor behavior, including invasion, proliferation, and resistance to treatment [94].

In the context of immune cells, Extracellular Vesicles (EV) can negatively influence dendritic cells (DCs) by impairing their antigen presentation abilities. Tumor-derived EVs, rich in miRNAs such as miR-203 and miR-212-3p, compromise DC functions and hamper the immune response [95,96]. This leads to an immuno-suppressive microenvironment within the tumor. miR-210 downregulation in pancreatic cancer enhances the infiltration of CD8+ T cells into the tumor, promoting a more robust immune response against cancer. miR-210 also causes inhibition of the M2 polarization of tumor-associated macrophages (TAMs) within the tumor, which leads to an immunotolerant environment [97]. Another microRNA miR-506 was found to promote the M1 reprogramming of TAMs and promote antitumor immunity [98].

Additionally, several other miRNAs have been implicated in regulating the immune response in pancreatic cancer. For instance, the miR-216 cluster, consisting of miR-216a-3p, miR-216a-5p, miR-216b-3p, and miR-216b-5p, is under-expressed in PDAC samples compared to normal pancreatic tissues. These miRNAs have an anti-tumorigenic effect, reducing tumor cell aggressiveness. Furthermore, miRNAs like miR-30c-2-3p, miR-148a-3p, miR-216b-5p, and miR-1827 influence the production of cytokines, such as IL-6 and CXCL-12, which play crucial roles in immune regulation [99]. Dysregulation of these miRNAs in pancreatic cancer has been linked to immune modulation and tumor progression. Another important factor that miRNA targets to cause immune suppression is the transcription factor BHLHE40, which affects cytokine production in T cells and macrophage proliferation [100]. Low expression of BHLHE40 is associated with an enrichment of various immune cell types, indicating its role in immune suppression and immune escape in pancreatic cancer. miR-15a-5p is known to target BHLHE40 and the downregulation of miR-15a leads to an immune-suppressive microenvironment in pancreatic cancer [101].

In summary, miRNAs within the pancreatic cancer microenvironment and their influence on immune infiltration are complex and multifaceted. They affect various aspects of the disease, including immunomodulation. Understanding these complex networks, which are essential for immune escape, is critical for developing targeted therapies and improving the treatment of pancreatic cancer.

**Table 1:** List of dysregulated miRNAs in pancreatic cancer, along with their targets.

**miRNA**	**Dysregulation**	**Targets**	**References**
miR-15a	Downregulated	***WEE1, CHK1, BMI-1, BCL2, Yap-1, and DCLK1, WNT3a, FGF7*** Cellular pathways, proliferation, EMT	[35,37,38,102]
miR-142	Downregulated	***FAK, MMP9, PIK3CA, HIF-1α*** Migration, angiogenesis, invasion	[40,41]
miR-145	Downregulated	***TGF-β receptor, angiopoietin-2 and SMAD2, MUC13, KRAS, RREB1*** Proliferation, migration, invasion, RAS signaling, angiogenesis	[42,43,103]
miR-373	Downregulated	***CCND2*** Propagation, migration, invasion, chemosensitivity to gemcitabine	[104,105]
miR-Let-7	Downregulated	***KRAS, STAT3, IGF2BP, and HMGA1/HMGA2, SOX13*** Progression and invasion	[106,107]
miR-141	Downregulated	***MAP4K4*** Proliferation and invasion	[108]
miR-34	Downregulated	***Snail1, Notch1*** Progression and invasion	[103,109]
miR-506	Downregulated	***STAT3, PIM3*** Polarization of M2-like macrophages, promotes antitumor immune response, overcomes immunotherapy resistance	[108]
miR-409	Downregulated	***GAB1*** *Cell cycle progression, migration, invasion*	[110]
miR-96	Downregulated	***NUAK1, KRAS*** Antiproliferative, Proapoptotic and Antimetastatic properties	[111,112]
miR-217	Downregulated	***Tpd52l2, ATAD2*** PIK3CA/AKT signaling pathways, inactivation of the AKT signaling pathway	[113,114]
miR-873	Downregulated	***PLEK2, KRAS*** PI3K/AKT pathway	[115,116]
miR-33a	Downregulated	***AMPK, METTL3, RAP2A*** MTOR signaling, EMT, metabolic reprogramming	[117,118,119]
miR-198	Downregulated	***MSLN, OCT-2, PBX-1, VCP*** Growth and metastases	[120]
miR-433	Downregulated	***GOT1*** Proliferation, metabolic reprogramming	[121]
miR-21	Upregulated	***PDCD4, Timp3, PTEN, RECK, Spry2*** MAPK/ERK and PI3K/AKT signaling pathways.	[75,122,123,124]
miR-221	Upregulated	***RB1, TIMP-2, KIT, CDKN1B, RUNX2 and BCL2*** 5-fluorouracil resistance, cell proliferation	[62,125,126]
miR-155	Upregulated	***TP53INP1*** Apoptosis	[127]
miR-27	Upregulated	***BTG2*** Wnt/β-catenin pathway	[128,129]
miR-196a	Upregulated	***NFKBIA*** Proliferation, migration	[130]
miR-194	Upregulated	***PD-L1, DACH1*** Anti-tumor immunity, progression	[131,132]
miR-212	Upregulated	***patched-1, Rb1*** Hedgehog signalling, cell cycle progression	[59,133]
miR-29a	Upregulated	***TTP*** EMT, Wnt/B-catenin	[134]
miR-191	Upregulated	***HIF-1, USP10*** Cell cycle progression	[135,136]
miR-23a	Upregulated	***FOXP2, TGFBR2, TFPI-2*** Proliferation and invasion	[71,137,138]

The table above summarizes a few of the miRNAs that are most commonly dysregulated in pancreatic cancer. However, there are several dysregulated miRNAs that have been shown to be downregulated in some studies and upregulated in a few others. For instance, miR-506 is upregulated in pancreatic cancer and has been shown to have oncogenic roles but some studies have also shown it to be downregulated and have tumor-suppressive roles [139,140]. Such inconsistencies could stem from diverse patient cohorts and limited sample size. Therefore, further investigation of such dysregulated miRNAs is crucial to ascertain their role as potential biomarkers. 

## 3. Potential Use of miRNA-Based Therapeutics for Pancreatic Cancer: Key Features and Limitations

Since miRNAs play a crucial role in regulating diverse cellular pathways that can contribute to tumor proliferation, invasion, and metastasis, microRNA (miRNA)-based therapies are emerging as a new strategy for cancer treatment due to their potential in the battle against various cancers, with a particular focus on pancreatic cancer. This multifaceted, miRNA-based therapeutic approach intends to re-establish the physiological expression and activity of miRNA in tumor cells. This strategy encompasses three key aspects: replenishing the tumor-suppressive miRNAs which are down-regulated in cancer cells, suppressing the activity of overexpressed miRNAs, and modifying miRNAs or using them in combination with other drugs to treat pancreatic cancer [141].

### 3.1. Restoration of Tumor-Suppressive miRNAs

One approach to miRNA-based cancer therapy involves replenishing the diminished levels of tumor-suppressor miRNAs. These miRNAs are designed to restore the natural balance that inhibits cancer progression. By introducing double-stranded miRNA mimics or viral vectors (adenoviral, lentiviral and retroviral vectors) expressing miRNAs, researchers aim to restore or enhance the tumor-suppressive functions of these miRNAs. These synthetic miRNAs can form an RISC complex and inhibit the target mRNAs, effectively reining in cancer cell growth and spread [25]. In the context of pancreatic cancer, where traditional treatment options are limited, this strategy provides an exciting avenue for improving therapeutic outcomes. 

For example, miR-34b, a p53-regulated miRNA is reduced in pancreatic cancer, but shows promise when overexpressed, leading to inhibited cell growth, cell cycle arrest, and enhanced sensitivity to chemotherapy through the repression of oncogenic Smad3 [142,143]. Similarly, the enforced expression of miR-34a (transfection of pre-miR-34a) was observed to target anti-apoptotic protein Bcl-2, c-Myc, Cyclin D1, E2F3 and Notch signaling pathway, causing the suppression of cancer cell proliferation, metastasis and invasion [144].

Han et al. demonstrated that the overexpression of miR-455-3p hinders the progression of pancreatic cancer by suppressing the Wnt/β-catenin signaling pathway mediated by the transcriptional co-activator with the PDZ-binding motif (TAZ). Additionally, it enhances apoptosis in pancreatic cancer cells by modulating the expression of apoptosis-related proteins such as Bcl-2 and Bax [145]. The ectopic expression of miR-15a was also found to cause cell cycle arrest and inhibit PDAC cell proliferation and EMT via the down-regulation of Bmi-1 expression [38]. Similarly, restoring the intracellular levels of miR-15b and miR-16 caused a reduction in Bcl-2 protein levels in activated pancreatic stellate cells (PSCs) and induced apoptosis [146]. Kent et al. reported that the viral-mediated transduction of miR-143/145 inhibits tumorous growth in pancreatic cancer cells [147]. Furthermore, the adenovirus-mediated delivery of miR-143 demonstrated an inhibitory effect on pancreatic cancer cells, effectively blocking metastasis [148].

miR-205 is downregulated in pancreatic cancer and, in an in vitro model, the overexpression of miR-205 decreased the expression of both general stemness markers such as OCT-3/4 and NANOG, and more specific pancreatic CSC markers such as CD44 and ALDH1 [90]. In another study by Srivastava et al., the restoration of miR-150 was found to significantly hinder the malignant potentials and growth of pancreatic tumor cells [149]. The re-expression of another downregulated miRNA, miR-137 significantly blocked the migration and invasion of pancreatic cancer cells by down-regulating MRG domain binding protein (MRGBP), which has been documented to be upregulated in malignant tumors [150]. 

Alternatively, treatment with trichostatin A, a chemotherapeutic drug, has been demonstrated to lead to the overexpression of several miRNAs that are typically suppressed in pancreatic cancer cell lines including miR-29a, miR-29b, miR-103, miR-107, and miR-320. Notably, the enforced expression of miR-107 in MiaPACA-2 and PANC-1 cells had a dual impact, inhibiting cell proliferation in vitro and also repressing the suspected target of miR-107, cyclin-dependent kinase 6 [150]. 

### 3.2. Suppressing miRNAs with Oncogenic Function

Another key aspect of miRNA-based therapies in pancreatic cancer focuses on the suppression of miRNAs with oncogenic function, miRNAs that are overexpressed in cancer cells and promote tumor growth and progression by targeting the suppressors of various key cellular pathways and modulating the tumor microenvironment. Downregulating miRNAs with oncogenic function has been shown to inhibit cell proliferation and induce apoptosis. Researchers have developed multiple ways to inhibit the activities of miRNAs with oncogenic functions such as the application of antisense anti-miR oligonucleotides (AMO), locked nucleic acid (LNA), miRNA antagomirs, and miRNA sponges. 

AMOs are chemically modified single-stranded RNA molecules, typically consisting of 17–22 nucleotides. They are synthetically designed to complement a specific miRNA of interest. The primary function of AMOs is to bind to the mature complementary miRNA with higher affinity, consequently inhibiting its interaction with specific mRNA targets [25]. A specific example of a modified AMO is Locked Nucleic Acid (LNA). LNA-modified antisense oligonucleotides offer distinct advantages as they exhibit higher thermal stability, greater solubility in aqueous environments, increased metabolic stability, and enhanced affinity to their target miRNA molecules, rendering them well-suited for in vivo delivery [151]. Anti-miRs have sequences that are complementary to the leading strand of the target miRNA. As a result, they can inhibit the activity of miRNAs with oncogenic function by binding to the seed region or by interfering with miRNA biogenesis. This approach represents a novel and promising way to combat pancreatic cancer at its core by disrupting the molecular mechanisms that fuel its growth [152].

For example, Griveau et al.’s in vivo study showed that LNA-modified antisense oligonucleotides effectively silenced overexpressed miR-21 in glioblastomas, leading to a significant reduction in cell viability and an increase in intracellular caspase levels [153]. Another study by Ma et al. reported that the inhibition of miR-10b using antagomirs effectively curbed metastasis in a mouse tumor model, simultaneously reducing miR-10b levels and promoting the expression of a functionally crucial miR-10b target, HOXD10. A low level of HOXD10 promotes the invasion and metastasis of cancer cells [154]. Similarly, another study exhibited impeded metastases in pancreatic cancer cells and primary human tumors upon the silencing of miR-10a. Suppressing miRNAs with oncogenic function, like miR-1976, using antagomiRs could sensitize PDAC cells to chemotherapeutic agents [155].

miRNAs with oncogenic function can also play a key role in maintaining cancer stem cells and tumor microenvironment. miR-135b was found to be upregulated in CD44+/CD24+/EpCAM+ CSCs. Inhibiting miR-135b with an antisense oligonucleotide (anti-miR-135b) decreased the expression of stemness markers such as NANOG, ALDH1, SOX2, and OCT-4 [86]. Recently, polycationic amphiphilic cyclodextrin (PCX) nanoparticles were used to co-deliver anti-miR-210 and siKRASG12D, which effectively delayed primary tumor growth by ~60% in pancreatic cancer. This codelivery also significantly decreased the collagen produced by activated PSCs, causing a reduced activation of PSCs and their interaction with stroma. Interestingly, the downregulation of miR-210 in the tumors further enhanced the infiltration of CD8+ T cells, possibly via T cell differentiation [97]. Additionally, exosomes derived from M2 TAMs containing miR-365 play a significant role in enhancing chemoresistance in pancreatic cancer [156]. 

Inspired by natural circular RNA (circRNA) acting as endogenous miRNA sponges, a novel artificial circRNA sponge has been engineered that can serve as an exogenous miRNA inhibitor and bind to mature miRNA. Notably, a single circRNA can regulate one or more miRNAs via distinct miRNA binding sites within its circular sequence [157]. In a study by Liu et al., circRNA sponges designed for miR-21 and integrated into a circular sponge-producing vector exhibited increased efficacy in suppressing their miRNA targets in lung cancer cells [158]. Furthermore, the transfection of the circRNA targeting miRNA-21 was shown to impede the proliferation of gastric cancer cells by inducing apoptosis and inducing global changes in protein expression via the modulation of miR-21-5p/RUNX1 axis [159]. 

Additionally, emerging miRNAs, such as miR-200a, miR-20a, miR-224, and miR-486, offer new possibilities for therapeutic interventions, playing roles as both oncogenic and tumor-suppressive agents. 

These findings underscore the diverse potential of miRNA-based therapies in tackling pancreatic cancer by targeting specific dysregulated miRNAs.

### 3.3. Combination Therapy

Variations in miRNA expression have been observed in a range of drug-resistant pancreatic cancer cells, underscoring the significance of miRNAs in the context of drug-based treatments for pancreatic cancer. 

Several tumor-suppressor miRNAs play an instrumental role in the reversal of 5-FU resistance in pancreatic cancer. The ectopic expression of miR-494 effectively restrains tumor cell proliferation, invasion, and resistance to 5-FU by directly targeting SIRT1 and c-Myc [160]. MiR-138-5p possesses the ability to target vimentin and sensitize pancreatic cancer cells to 5-FU [161]. Furthermore, miR-34a plays a significant role in making pancreatic cancer cells more responsive to chemotherapy and radiotherapy, ultimately leading to the suppression of cancer growth and the induction of cell apoptosis by inhibiting the expression of Bcl-2 and Notch signaling pathways [143]. Importantly, the in vitro introduction of miR-34a has been shown to markedly enhance the anticancer effects of 5-FU, further highlighting the therapeutic potential of miRNAs in sensitizing pancreatic cancer cells. 

Similarly, several tumor-suppressor miRNAs play a key role in sensitizing pancreatic cancer cells to Gemcitabine treatment. In a study by Hu et al., miR-373-3p was found to reduce the expression of Cyclin D2, resulting in increased sensitivity to gemcitabine and the suppression of growth in gemcitabine-resistant pancreatic cancer cells [104]. miR-663a, miR-142-5p, and miR-145 were also shown to enhance the chemosensitivity towards gemcitabine. 

The miR-17-92 cluster functions to mitigate stemness and GEM resistance in pancreatic cancer stem cells by acting on the NODAL/ACTIVIN/TGF-b1 pathway [162]. Additionally, the restoration of miR-34 in human pancreatic cancer BxPC3 and MiaPaCa2 cells induces apoptosis and enhances sensitivity to GEM by inhibiting the expression of Notch1, Notch2, and Bcl-2 [85]. Furthermore, miR-210 overexpression inhibits pancreatic cancer growth and reverses gemcitabine resistance by directly targeting the multi-drug efflux transporter ABCC5, a crucial factor in drug resistance in various cancers [163]. 

miRNAs also play a crucial role in sensitizing several other drug resistances. For instance, with cisplatin, a potent chemotherapy drug, inhibiting miR-1180 can enhance cisplatin-induced apoptosis, thereby countering resistance [164]. Increasing the expression of miR-100 also significantly increased cisplatin sensitivity by reducing FGFR3 mRNA levels [165]. MiR-374b’s down-regulation contributes to acquired cisplatin resistance, but its ectopic expression in resistant cell lines can effectively reduce drug resistance [166]. The overexpression of miR-137 was reported to enhance doxorubicin’s effect on reducing pancreatic cancer cell survival by inhibiting autophagy mediated by ATG5 [167]. Additionally, using PL-1/miR-9 nanoparticles significantly enhanced the antitumor effect of doxorubicin by downregulating eIF5A2 [168]. 

FOLFIRINOX, a combination of the chemotherapeutic drugs Fluorouracil, Oxaliplatin, and Irinotecan, is known to be highly effective in advanced unresectable PDAC, boasting a response rate exceeding 30%. However, 75% of patients experience severe toxic side effects, underscoring the necessity for biomarkers to guide treatment decisions. Functional biological analyses reveal that miR-1307 plays a crucial role in the dynamics of drug response, particularly affecting sensitivity to platinum-containing regimens within the FOLFIRINOX regimen. Notably, miR1307 influences the expression of CLIC5, a protein involved in ion transport within cellular compartments, and its impact appears to be linked to platinum-induced DNA damage. These findings suggest that miRNAs like miR-1307 may serve as potential biomarkers when tailoring FOLFIRINOX-based treatments, increasing their efficacy and minimizing unnecessary toxicities in PDAC patients [169].

Hence, the combination of miRNAs and conventional drug therapies could refine treatments, enhancing their precision and effectiveness. This synergy between microRNAs and drugs not only addresses chemoresistance but also offers a new avenue to enhance outcomes for individuals battling pancreatic cancer, signifying a path towards more successful cancer treatments.

### 3.4. Modification of miRNAs to Treat Pancreatic Cancer

MRNA modification represents a novel and promising approach in cancer therapeutics, particularly in the context of pancreatic ductal adenocarcinoma (PDAC). This approach involves enhancing the stability and efficacy of miRNAs for more effective treatments. One key example is miR-15a, which plays a role in inhibiting cell proliferation and regulating cell cycle control in PDAC. To improve its performance, a modified version, 5-FU-miR-15a, was developed by replacing the uracil residues of the guide strand miRNA with 5-fluorouracil (5-FU). The rationale behind this modification is to combine the therapeutic potential of 5-FU with the tumor-suppressive function of miR-15a. miR-15a has several important targets, such as Wee-1, Chk-1, Bmi-1 and Yap-1, which are significantly overexpressed in PDAC. Notably, 5-FU-miR-15a was shown to maintain its specificity for these targets. Furthermore, once the modified miRNA is degraded within the tumor cells, the released 5-FU molecules were found to exert an additional tumor-killing effect, enhancing the overall therapeutic outcome [170]. This innovative concept was successfully demonstrated in colorectal cancer and has been shown to have great potential for PDAC treatment. Notably, 5-FU-miR-15a was found to be potent in inhibiting PDAC metastatic tumor growth and sensitizing PDAC to gemcitabine in vivo, showcasing its therapeutic promise [38]. 5-FU-miR-15a was also shown to significantly inhibit the expression of Yap1, Bcl2, Il6, and Mmp9, both alone and during treatment with TGFβ1 in murine and human Pancreatic Stellate Cells, suggesting the reduced proliferation and migration of pancreatic stellate and cancer cells [171].

Moreover, the strategy of incorporating 5-FU into miR-15a offers a platform technology for the creation of more effective miRNA-based therapeutics. This approach not only enhances target specificity but also maintains efficacy without the need for delivery vehicles, both in vitro and in vivo. The key advantage of this modification is its ability to be delivered to cancer cells, vehicle-free, which may potentially overcome the challenge of vehicle-associated toxicities (toxicity associated with systemic delivery by lipid-based nanoparticles). This technology platform has broad potential for the development of miRNA-based therapies, and its success is evident in terms of it retaining target specificity, enhancing potency, and increasing intracellular stability, making 5-FU-modified miRNA mimetics promising candidates for cancer treatment in the low nanomolar range [38]. Another such approach, which shows a promising therapeutic outcome, is the Gemcitabine modification of miR15a. Replacing cytidine of the guide strand miRNA with gemcitabine, Gem-miR15a and Gem-miR194 was shown to have significant effects on the sensitization og pancreatic cancer to chemotherapy, as well as increased efficacy compared to its counterpart, unmodified miRNA. However, the impact of Gem-miR-194 was not as significant as the effects of Gem-miR-15a in the in vivo context [13]. In summary, miRNA modification with 5-FU or other chemotherapeutic drugs like Gemcitabine represents an innovative avenue in miRNA-based cancer therapy, paving the way for more effective and targeted treatments with broad applicability in diverse cancer types.

Further investigations into miRNA modifications have shown that the stability of miRNAs can be influenced by various factors, including the addition of uridine to the miRNA 3′ end and the differential processing of pre-miRNAs to mature miRNAs based on secondary structures. These modifications can impact miRNA’s stability, target specificity, and therapeutic efficacy.

### 3.5. Limitations of miRNA-Based Therapeutics

As we discussed, miRNA-based therapeutics show great potential in the field of cancer treatment. However, there are a lot of challenges in the development and delivery of miRNAs. The primary goal of any therapy is to achieve specificity without any off-target effects, which is the biggest challenge in the field of miRNA therapeutics. The next biggest limitation is the efficient, targeted delivery of miRNA molecules. miRNAs face difficulty in crossing the cell membrane and might therefore be degraded. 

Currently, viral or non-viral vectors are being used for miRNA delivery. Nanoparticles also show great promise in the delivery of miRNAs or antagomiRs. Chemical modifications of the nucleic acid strands have been shown to increase the stability of these molecules and also, in certain cases, remove the need for vehicle-mediated delivery. It is important to consider the immunological response of these molecules, as well as the optimal dosing to establish a therapeutic effect. Population-dependent variability in miRNA expression patterns adds an extra layer of complexity to the field. 

However, miRNA-based therapy has shown immense promise in the treatment of cancer by targeting multiple targets at once. Ongoing research efforts aim to potentially overcome these limitations and establish a miRNA-based therapy for cancer.

## 4. Conclusions

Pancreatic cancer is among the most formidable of all cancers, predicted to exceed most cancers and become the second leading cause of cancer-related death by 2030. The genetic heterogeneity and late diagnosis of the disease severely retard efforts to manage pancreatic cancer. Thus, there is a pressing need to develop novel therapies and biomarkers for the early detection of pancreatic cancer. 

In this regard, miRNAs have emerged as promising players, which can aid in the early detection and treatment of pancreatic cancer. The distinctive miRNA expression profiles not only allow for the establishment of cancer signatures but can also serve as essential therapeutic targets. 

In this review, we showed the differential regulation of microRNAs in the context of pancreatic cancer and explored how the targeted modulation of miRNAs via the delivery of miRNA mimics, anti-miRNA therapies and modifications in the miRNA could have profound effects on the disease by regulating multiple signaling pathways at once. miRNA treatment, alone or in conjunction with other chemotherapeutic drugs, not only inhibits cancer progression but has also been shown to overcome chemoresistance to various chemotherapeutic drugs, thereby improving patient outcomes. Further, the modifications in miRNA that bypass the need for vehicle-mediated delivery have overcome the limitation of the vehicle eliciting an immune response or vehicle-mediated toxicity. 

In conclusion, the field of miRNA-based therapeutics is a new and exciting area in the quest against pancreatic cancer. While there are still a lot of hurdles due to the tumor-suppressive microenvironment of pancreatic cancer, as well as its genetic heterogeneity, tremendous research efforts and progress have been made in terms of biomarkers and early diagnosis. As a result, these efforts have established a solid foundation on which to develop novel miRNA-based early detection and treatment strategies, to improve the future clinical management of pancreatic cancer and quality of life.
